# Modeling Parkinson’s Disease With the Alpha-Synuclein Protein

**DOI:** 10.3389/fphar.2020.00356

**Published:** 2020-04-23

**Authors:** Mónica Gómez-Benito, Noelia Granado, Patricia García-Sanz, Anne Michel, Mireille Dumoulin, Rosario Moratalla

**Affiliations:** ^1^ Cajal Institute, Consejo Superior de Investigaciones Científicas (CSIC), Madrid, Spain; ^2^ CIBERNED, Instituto de Salud Carlos III, Madrid, Spain; ^3^ UCB Biopharma, Neuroscience TA, Braine L'Alleud, Belgium; ^4^ Centre of Protein Engineering, InBios, University of Liege, Liège, Belgium

**Keywords:** Lewy body pathology, α-Syn pre-formed fibrils, α-Syn AVV-viral particles, prion-like propagation, Braak hypothesis, α-Syn aggregation

## Abstract

Alpha-synuclein (α-Syn) is a key protein involved in Parkinson's disease (PD) pathology. PD is characterized by the loss of dopaminergic neuronal cells in the substantia nigra pars compacta and the abnormal accumulation and aggregation of α-Syn in the form of Lewy bodies and Lewy neurites. More precisely, the aggregation of α-Syn is associated with the dysfunctionality and degeneration of neurons in PD. Moreover, mutations in the *SNCA* gene, which encodes α-Syn, cause familial forms of PD and are the basis of sporadic PD risk. Given the role of the α-Syn protein in the pathology of PD, animal models that reflect the dopaminergic neuronal loss and the widespread and progressive formation of α-Syn aggregates in different areas of the brain constitute a valuable tool. Indeed, animal models of PD are important for understanding the molecular mechanisms of the disease and might contribute to the development and validation of new therapies. In the absence of animal models that faithfully reproduce human PD, in recent years, numerous animal models of PD based on α-Syn have been generated. In this review, we summarize the main features of the α-Syn pre-formed fibrils (PFFs) model and recombinant adeno-associated virus vector (rAAV) mediated α-Syn overexpression models, providing a detailed comparative analysis of both models. Here, we discuss how each model has contributed to our understanding of PD pathology and the advantages and weakness of each of them.

**Significance:**

Here, we show that injection of α-Syn PFFs and overexpression of α-Syn mediated by rAAV lead to a different pattern of PD pathology in rodents. First, α-Syn PFFs models trigger the Lewy body-like inclusions formation in brain regions directly interconnected with the injection site, suggesting that there is an inter-neuronal transmission of the α-Syn pathology. In contrast, rAAV-mediated α-Syn overexpression in the brain limits the α-Syn aggregates within the transduced neurons. Second, phosphorylated α-Syn inclusions obtained with rAAV are predominantly nuclear with a punctate appearance that becomes diffuse along the neuronal fibers, whereas α-Syn PFFs models lead to the formation of cytoplasmic aggregates of phosphorylated α-Syn reminiscent of Lewy bodies and Lewy neurites.

## Parkinson's Disease

Parkinson's Disease (PD) is the second most common neurodegenerative disorder after Alzheimer's disease. Currently, PD affects 1%–2% of people over the age of 60 years, rising to 4% at age 80 years (Rizek et al., 2016). PD is mainly characterized by the progressive loss of dopaminergic neurons of the substantia nigra that project to the striatum (Xu and Pu, 2016). The deficiency of dopamine in the striatum leads to the development of the classic motor symptoms of PD, including bradykinesia, resting tremor, muscular rigidity, and postural instability. In addition to motor symptomatology, non-motor manifestations, such as autonomic dysfunction, olfactory impairment, mood disorders (i.e., depression and anxiety), cognitive deficits, or sleep disturbances are also frequently present in PD. Most of these symptoms appear even before the motor symptoms and have a serious impact on the quality of life of patients (Kalia and Lang, 2015; Schapira et al., 2017). Because the PD motor symptoms emerge when the striatal dopamine levels have decreased by 60%–80% (Dauer and Przedborski, 2003), the study of PD non-motor symptoms is important to identify early biomarkers as well as targets to develop disease-modifying therapies that slow or prevent the progression of neurodegeneration.

The neuropathological mechanisms underlying non-motor symptoms of PD are still poorly understood, but growing evidence suggests that the evolution of these symptoms may arise from the disruption of both dopaminergic and non-dopaminergic systems, and the involvement of diverse structures outside the nigrostriatal system (Jellinger, 2017; Schapira et al., 2017). Besides the dopamine, the further key neurotransmission systems that have been described to be involved in the pathogenesis of PD are the noradrenergic system of locus coeruleus, the serotonergic system of the dorsal raphe nuclei, and the cholinergic system of the nucleus basalis of Meynert and the pedunculopontine nucleus (Qamar et al., 2017). Since the relative contribution of each of these pathways to motor and non-motor symptoms has only been partially explored, additional research is needed to fully understand their involvement in the clinical and pathological features of the disease.

The neuropathological hallmark of PD is the abnormal accumulation and aggregation of alpha synuclein protein (α-Syn) in form of Lewy bodies and Lewy neurites (Xu and Pu, 2016). It is well established that pathological aggregation of α-Syn is a common feature of several neurodegenerative diseases including PD, dementia with Lewy bodies (DLB) and multiple system atrophy (MSA), that are collectively referred as synucleinopathies (Goedert et al., 2017). α-Syn is a protein with remarkable conformational plasticity since it can adopt a wide range of structural conformations (oligomers, protofibrils and fibrils; Deleersnijder et al., 2013; Mehra et al., 2019). Each α-Syn conformation displays distinct properties in terms of neurotoxicity, stability and seeding and propagation ability. It has been proposed that the existence of structurally distinct α-Syn assemblies or α-Syn “strains” may contribute to explain the clinical-pathological heterogeneity among synucleinopathies and help to develop strain-specific medications (Melki, 2015; Peelaerts et al., 2015; Candelise et al., 2019).

Parallel to deficits in both dopaminergic and non-dopaminergic neurotransmission systems, the appearance of PD non-motor symptoms is also attributed to the sequential development of Lewy bodies in different brain regions, including the olfactory bulb, the dorsal motor nucleus of the vagal nerve, locus coeruleus, raphe nucleus, basal nucleus of Meynert and pedunculopontine nucleus (Braak et al., 2003; Braak et al., 2004; Schindlbeck and Eidelberg, 2019). Both hypotheses are not mutually exclusive, since sequential distribution of Lewy bodies through the brain may alter various neurotransmission pathways that may be the basis of non-motor manifestations. To date, the etiology of PD remains unknown, but cumulative evidence suggests that the presence of intraneuronal inclusions of α-Syn affects the functional integrity of neurons, ultimately causing their death. It has been demonstrated that α-Syn aggregates can induce neuronal toxicity leading to neuronal death by multiple mechanisms, including mitochondrial dysfunction, lysosomal impairment, membrane disturbance, endoplasmic reticulum stress, and synaptic dysfunction (reviewed in Roberts and Brown, 2015; Xu and Pu, 2016; Zhang et al., 2018; Zhang et al., 2019). However, although the Lewy pathology is commonly observed in PD, there remains much debate over whether α-Syn aggregation is a key feature for the development and progression of the disease. Two observations contribute to this debate: first, there is solid evidence that not all cases of parkinsonism are characterized by the presence of α-Syn inclusions—several studies have reported that PD patients carrying familial mutations in *Parkin* gene, and some of those with the *LRRK2* G2019S mutation, show neuronal degeneration but do not develop Lewy bodies (Gaig et al., 2009; Johansen et al., 2018) and, second, postmortem analysis reflect that Lewy bodies and Lewy neurites may be present in the absence of clinical PD symptoms (Parkkinen et al., 2005). Strengths and limitations of the evidence that correlate the aggregation of α-Syn with the progression of PD pathology will be addressed throughout the review.

## The Role of Alpha-Synuclein in PD Pathology

α-Syn is a small protein encoded by the *SNCA* gene that is abundantly expressed in the presynaptic terminals of the central nervous system. The exact function of α-Syn remains largely unknown, although mounting evidence supports the notion that α-Syn is involved in synaptic plasticity and neurotransmitter release (Burré et al., 2010; Venda et al., 2010). Likewise, recent studies have shown that neuronal/synaptic activity regulates dynamically the physiological release of endogenous α-Syn, so an elevated neuronal activity increases the release of α-Syn (Yamada and Iwatsubo, 2018). Several lines of evidence demonstrate the pathogenic role of α-Syn in PD: 1) point mutations (A30P, E46K, H50Q, G51D, A53T, and A53E) and duplication or triplication of the *SNCA* gene cause autosomal dominant forms of PD (Polymeropoulos et al., 1997; Zarranz et al., 2004); 2) polymorphic variants of the *SNCA* gene constitute an important risk factor for developing idiopathic PD (Nalls et al., 2014); and 3) α-Syn is the major component of Lewy bodies (Spillantini et al., 1997; Wakabayashi et al., 2013).

Under normal conditions, native α-Syn exists in a dynamic equilibrium between unfolded monomers and α-helically folded tetramers with a low propensity to aggregation (Lashuel et al., 2013). The decline of the tetramer:monomer ratio and the consequent increase in the level of α-Syn unfolded monomers favor its aggregation (Nuber et al., 2018). The aggregation process of α-Syn involves a conformational change whereby it adopts a β-sheet-rich structure that facilitates its aggregation into oligomers, protofibrils, and insoluble fibrils that finally accumulate in Lewy bodies. There is an intense debate about what α-Syn species are cytotoxic. Although both oligomeric and fibrillar species of α-Syn have been shown to be toxic, recent studies suggest that oligomers and protofibrils, forming during the initial stages of the aggregation process, are the potent neurotoxic species causing cell death in PD. Conversely, α-Syn fibrils appear to be the most efficient species at propagating, thus contributing to the spread and progression of the disease (Alam et al., 2019; Mehra et al., 2019). Most of studies that confirm the pathogenic effects of different α-Syn assemblies have used in vitro formed species; so the extent to which these oligomers recapitulate the structure and properties of those found in brain tissue from PD patients remains unclear (Bengoa-Vergniory et al., 2017). Mutations, post-translational modifications, an imbalance between synthesis and degradation of α-Syn, and environmental factors influence the aggregation propensity of α-Syn. The A53T mutation was the first to be documented, and it is associated with an early-onset PD (Polymeropoulos et al., 1997). The E46K mutation predisposes to the development of severe parkinsonism with dementia and a large number of Lewy bodies that are widely distributed (Zarranz et al., 2004). Both mutations alter the α-Syn protein structure, which facilitates its aggregation (Li et al, 2001; Greenbaum et al., 2005; Tosatto et al., 2015).

α-Syn undergoes various post-translational modifications, such as phosphorylation, truncation, ubiquitination, and nitration. Phosphorylation of α-Syn at the serine 129 residue is one of the major pathological markers of PD; 90% of α-Syn is phosphorylated in the brain of patients with PD while only 4% of α-Syn is phosphorylated in healthy brains (Oueslati, 2016; Ghosh et al., 2017). However, there is a great controversy over whether phosphorylation has an active role in the α-Syn aggregation or if it is a response mechanism of cells to try to label and eliminate toxic species of α-Syn (Oueslati, 2016). Smith et al. (2005) proposed that phosphorylation of α-Syn promotes the formation of cytoplasmic inclusions in some cell culture models (Smith et al., 2005). Nevertheless, Oueslati et al. (2013) show that phosphorylation of α-Syn induced by polo-like kinase 2 has no effect on the aggregation and regulates α-Syn clearance via the lysosomal autophagy pathway (Oueslati et al., 2013). Additional lines of evidence show crosstalk between phosphorylation and α-Syn degradation. The inhibition of the ubiquitin-proteasome system (Chau et al., 2009) or the autophagy-lysosomal pathway (Machiya et al., 2010) induced a significant increase in phosphorylated α-Syn in human neuroblastoma, suggesting that phosphorylation regulates the α-Syn degradation.

## Propagation of Alpha-Synuclein: Evidence and Considerations

Although dopaminergic neurons of substantia nigra seem to be particularly vulnerable in PD, the examination of PD progression indicates that α-Syn pathology is not restricted exclusively to this region. In 2003, Braak et al. postulated the hypothesis that the progression of α-Syn pathology follows a specific caudo-rostral pattern through the central nervous system (Braak et al., 2003; Braak et al., 2004). These authors proposed that the two starting points of PD pathology were the olfactory bulb and the enteric nerves, and from them, the damage extends via the olfactory tract or the vagus nerve, respectively, to other brain regions. According to this theory, PD can be divided into six stages, and each of them is characterized by the development of α-Syn inclusions in specific brain areas, including dorsal motor nucleus of vagus nerve, raphe nuclei, magnocellular portions of reticular formation, locus coeruleus, substantia nigra, and cortex (Killinger and Kordower, 2019). The presence of these inclusion bodies causes a dysfunctionality of the cells, which is ultimately responsible for the development of the clinical symptoms associated with PD (Braak et al., 2003; Braak et al., 2004). Given that α-Syn is involved in neuronal plasticity, the functional consequences of its aggregation have been explored at both the presynaptic and postsynaptic level. A recent study has shown that neurons derived from human induced pluripotent stem cells (iPSC) of PD patients that express oligomer-forming α-Syn mutants (E46K and E57K) display a reduction in presynaptic protein levels, an impaired anterograde axonal transport, and structural abnormalities of the axonal and synaptic compartments (Prots et al., 2018). Moreover, other studies have shown that α-Syn plays a role in N-Methyl-D-aspartic acid (NMDA) receptor trafficking, suggesting that α-Syn has postsynaptic effects. In a transgenic mouse model expressing C-terminally truncated α-Syn (aa 1-120), an impaired hippocampal long-term potentiation due to alterations in dopaminergic transmission and plastic changes in the composition of NMDA receptors has been reported (Tofaris et al., 2006; Costa et al., 2012). Electrophysiological recordings after treating mouse brain slices with α-Syn oligomers show impaired synaptic transmission and long-term potentiation in the hippocampus (Martin et al., 2012; Diógenes et al., 2012). *In vivo* amperometry recordings in rodents injected with α-Syn PFFs in combination with AAV-mediated overexpression of α-Syn reveal a reduction in dopamine release and reuptake rates in the striatum (Thakur et al., 2017). Recently, an electrophysiological analysis from slices from α-Syn PFFs-injected mice has shown that α-Syn reduces NMDA receptor-mediated synaptic currents and impairs long-term potentiation in the striatal medium spiny neurons (Durante et al., 2019).

Moreover, Kordower et al. found Lewy body-like inclusions in embryonic grafted neurons in PD patients, suggesting that α-Syn can spread from the host to the graft neurons (Kordower et al., 2008). The α-Syn protein has been detected in cerebrospinal fluid, and blood plasma of both PD and healthy subjects (El-Agnaf et al., 2003) and, in addition, numerous *in vitro* studies have demonstrated that cultured neurons can secrete α-Syn and take it up from the extracellular space (Luk et al., 2009; Volpicelli-Daley et al., 2011; Reyes et al., 2015). All these findings suggest that pathological α-Syn acts as a prion-like protein that can propagate throughout the brain through cell-to-cell transmission mechanisms. Moreover, the treatment of cultured cells with recombinant α-Syn fibrils induces the aggregation of endogenous α-Syn in insoluble inclusions that resemble Lewy bodies. These data suggest that PFFs can seed the recruitment of endogenous α-Syn and induce its pathological conversion (Luk et al., 2009; Volpicelli-Daley et al., 2011). In addition to *in vitro* studies, in the last decade, several animal models of PD have been developed to study (*in vivo*) the ability of propagation of α-Syn protein. These models can be classified as (a) neural stem cell transplantation into transgenic mice expressing human α-Syn (Desplats et al., 2009), (b) administration of brain extracts derived from PD patients (Recasens et al., 2014) or α-Syn pre-formed fibrils (PFFs; Chung et al., 2019), and (c) AAV-α-Syn viral particles (Ulusoy et al., 2013; Ip et al., 2017). Among these, the PFFs and the AAV-α-Syn viral particle models, which are the subject of this review, are the most extensively used models to study the α-Syn pathology and propagation.

Although many studies support the prion-like nature of α-Syn and show that its propagation determines the temporal course of the disease, some observations have recently challenged this theory. The Braak staging is neither a proof nor an argument of the spreading hypothesis, it might be rather that certain subsets of neurons are affected by Lewy bodies much earlier than others and that, therefore, the intraneuronal lesions evolve sequentially (Walsh and Selkoe, 2016). Another clinical observation that has questioned the pathogenic spread hypothesis is that only a minority of grafted neurons in patients with advanced PD exhibited Lewy body-like inclusions, and their presence appeared to have little functional consequences for neurons that survived for long periods of time (Kordower et al., 2008; Cooper et al., 2009; Hallett et al., 2014). In this context, a new possibility raised is that a selective vulnerability of specific neuronal populations to certain adverse stimuli, such as neuroinflammation, contributes to the propagation of α-Syn (Walsh and Selkoe, 2016). Misfolded α-Syn can trigger the activation of microglia. Likewise, activated microglia can enhance the aggregation and spreading of α-Syn, creating a positive feedback loop between inflammation and α-Syn aggregation. Both phenomena are interconnected, and this interaction plays a key role in the pathogenesis of PD. Therefore, it remains unclear whether α-Syn aggregation is a cause or a consequence of inflammation. A recent study suggests that activation of microglia with toxic cytokine release, such as caspase-1 and calpains, plays a critical role in promoting the misfolding of native α-Syn and in the spread of misfolded α-Syn in PD (Olanow et al., 2019). The two proposed possibilities, the prion-like hypothesis and the selective vulnerability hypothesis, are not mutually exclusive, and a combination of both hypotheses might occur. It is noteworthy that in the PFFs models, microglial activation might appear as resulting from an immune reaction due to the inoculation of foreign α-Syn fibrils. Indeed, it has been shown that striatal injection of α-Syn PFFs but not monomers in WT mice can trigger neuro-inflammation by increasing peripheral immune cells infiltration in the CNS (Earls et al., 2019). Apparently, this immune reaction would precede the dopaminergic neurodegeneration (Harms et al., 2017) but, it cannot be excluded that the magnitude and the efficacy of the immune reaction can also modulate the spread of the pathology observed as it was recently suggested by (Earls et al., 2020).

Another crucial question that is not clear is whether the spreading of α-Syn is a driving factor for neuronal degeneration and progression of PD, or if it is an epiphenomenon that appears as a result of other alterations, such as lysosomal dysfunction (Killinger and Kordower, 2019). Despite the unequivocal evidence of the presence of Lewy body-like inclusions and the ability of α-Syn to propagate in animal models, why dopaminergic neurons of SNc should be particularly vulnerable to propagated aggregates of α-Syn remains uncertain. The lack of α-Syn deposits in some PD, patients (Gaig et al., 2009; Johansen et al., 2018) has led some authors to the belief that the presence and spread of Lewy-type aggregates are not sufficient to explain the dysfunction and loss of neurons and the development of parkinsonian symptoms.

Lysosomal dysfunction impairs the ability to remove toxic aggregates, which increases the probability of α-Syn aggregation and spreading (Klein and Mazzulli, 2018). α-Syn is degraded through the lysosome in physiological conditions, so some perturbations in lysosomal functions can affect the α-Syn levels (Martinez-Vicente et al., 2008). Likewise, α-Syn aggregates might impair the autophagic-lysosomal pathway function (Xilouri et al., 2013a), establishing a reciprocal relationship. The accumulation of α-Syn reduces lysosomal degradation capacity by disrupting hydrolases trafficking, such as of glucocerebrosidase 1 (GCase1), from the endoplasmic reticulum to the lysosome (Mazzulli et al., 2011; García-Sanz et al., 2017; García-Sanz et al., 2018). Currently, mutations in the *GBA1* gene are the main genetic risk factor for PD. The *GBA1* gene encodes for GCase1, a lysosomal enzyme responsible for degrading the lipid glucosylceramide into ceramide and glucose (Do et al., 2019). *GBA1* mutations result in reduced enzymatic activity that leads to the accumulation of glucosylceramide and of cholesterol and its esters in lysosomes, which can compromise lysosomal function and promote α-Syn aggregation, creating a bidirectional loop (Schapira, 2015). Previous studies from our laboratory have shown that fibroblasts derived from PD patients with the *GBA1* mutation accumulate cholesterol in lysosomes and present multilamellar bodies (García-Sanz et al., 2017; García-Sanz et al., 2018). Also, membrane structures resembling lysosomes and autophagosomes have been found in the inner architecture of Lewy bodies (Shahmoradian et al., 2019), suggesting that the alteration of these organelles might contribute to the formation of Lewy bodies.

In addition to *GBA1* mutations, numerous PD-related genetic variants have been identified in several genes involved in the autophagic-lysosomal pathway including *Parkin*, *PINK1*, *DJ-1*, *LRRK2*, *ATP13A2*, or *VPS35* (Klein and Westenberger, 2012). Some of these genes encode lysosomal enzymes/proteins (e.g. *ATP13A2*), whereas others correspond to proteins that are involved in the transport to the lysosome (e.g. *VPS35*), mitophagy (e.g. *Parkin*, *PINK1*, and *DJ-1*), or other autophagic-related functions (e.g. *LRRK2*; Gan-Or et al., 2015). Mutations in these genes are directly related to autosomal -dominant or recessive- PD forms or may contribute to increase PD susceptibility.

Propagation and aggregation of α-Syn is an important molecular mechanism that contributes to PD progression. However, this event might require other factors to promote the pathological development of the disease. Mitochondrial dysfunction, oxidative stress, failure of the lysosomal autophagy and ubiquitin-proteasome systems, and neuroinflammation have been recognized as potential triggers of the misfolding of α-Syn, the spread of pathology, and the progression of PD. Precisely, Lewy bodies are composed by fragmented organelles including mitochondria, lipid membranes, lysosomal structures as well as other proteins involved in the degradation systems such as ubiquitin and p62/SQSTM1, suggesting a potential role of damaged and disrupted organelles in the formation of α-Syn inclusions (Shahmoradian et al., 2019). All these factors depend on the age, genetic background, and environment to which each individual is exposed. Likewise, the misfolding of α-Syn and its consequent aggregation might cause several alterations, such as mitochondrial dysfunction, endoplasmic reticulum stress, impairment of protein clearance pathways, disruption of biological membranes, and synaptic dysfunction (reviewed in Roberts and Brown, 2015). Future research should focus on determining which events lead first to the development of pathology and understand how the different mechanisms involved interact with each other.

## Animal Models of PD Based on Alpha-Synuclein

Given the close relationship between α-Syn aggregation and PD pathology, a wide variety of PD animal models has been generated in recent years. This review includes a detailed analysis of models based on the inoculation of α-Syn PFFs and models based on overexpression of α-Syn by recombinant adeno-associated viral vectors (rAAV), giving special interest to the differences observed between these two models. These observations are based on results obtained by our group and which are in line with the findings of previous studies.

### Injection of α-Syn Pre-Formed Fibrils or Pathological Extracts

**Figure 1:**
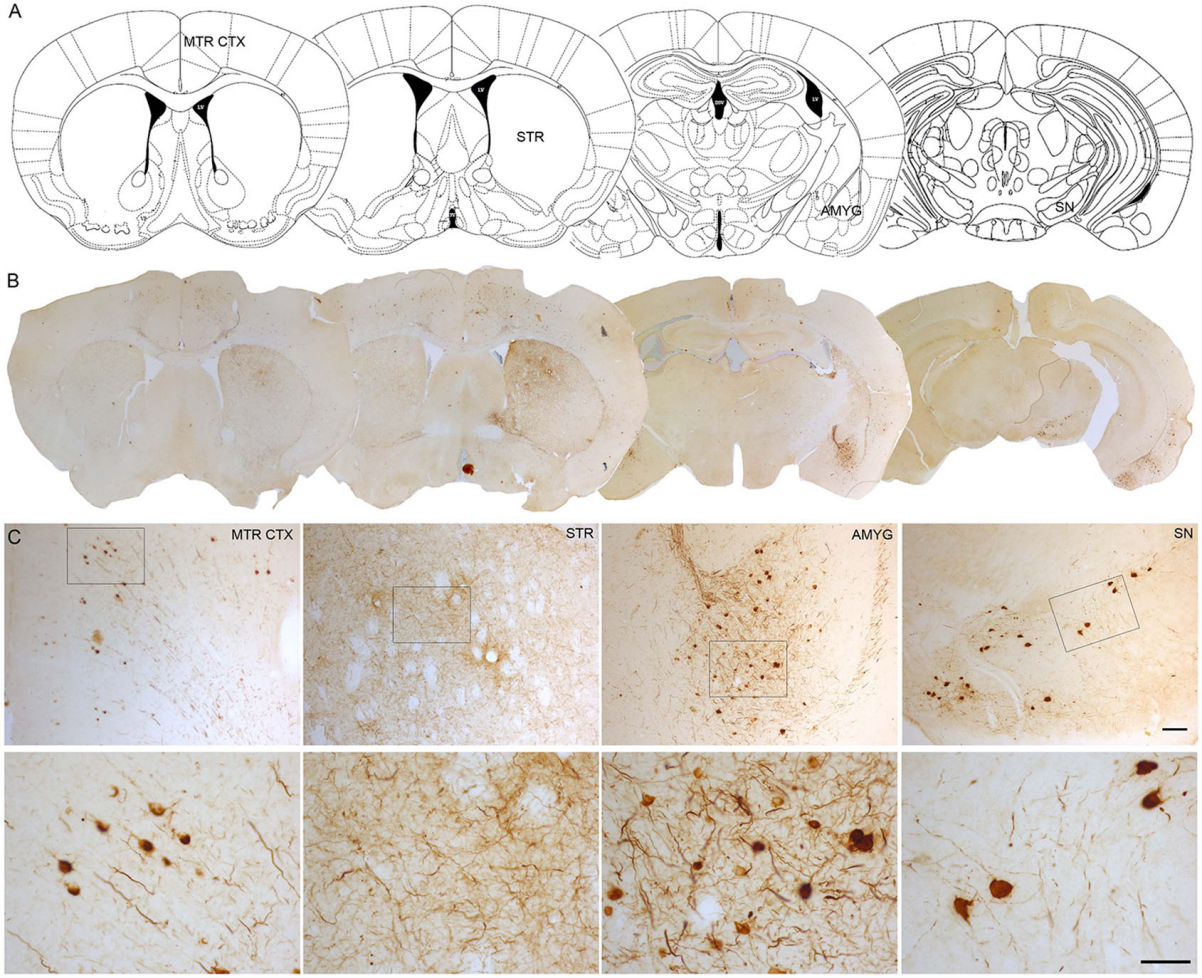
α-Syn aggregation 3 months after intrastriatal α-Syn PFFs injection. **(A, B)**. Representative photomicrographs of pα-Syn-stained sections of the motor cortex (Mrt Ctx), striatum (STR), amygdala (Amyg), and substantia nigra (SN). **(C)**. High magnification images show the presence of pα-Syn. Scale bar: 100 µm (**C**, upper panel) and 50 µm (**C**,lower panel). PFFs, pre-formed fibrils.

One of the approaches that have been developed to study the propagation of α-Syn is the intracerebral or systemic administration of either α-Syn pre-formed fibrils (PFFs) or brain extracts containing Lewy bodies and α-Syn derived from PD patients or transgenic mice exhibiting α-Syn pathology. PFFs are generated *in vitro* from recombinant α-Syn monomers. Subsequently, the aggregation of monomers into fibrils is induced and the fibrils are sonicated to generate short fibrils which after injection, trigger the aggregation, hyperphosphorylation and ubiquitination of endogenous α-Syn (Patterson et al., 2019). Intracerebral injection of α-Syn PFFs has been extended to rodents, both mice (Luk et al., 2012a; Masuda-Suzukake et al., 2013; Karampetsou et al., 2017; Milanese et al., 2018; Okuzumi et al., 2018; Patterson et al., 2019) and rats (Paumier et al., 2015; Harms et al., 2017; Thakur et al., 2017; Duffy et al., 2018b; Durante et al., 2019) and non-human primates (Shimozawa et al., 2017; Chu et al., 2019). PFFs have been mainly injected in the striatum (Luk et al., 2012a; Paumier et al., 2015; Karampetsou et al., 2017; Shimozawa et al., 2017; Duffy et al., 2018b; Milanese et al., 2018; Okuzumi et al., 2018; Chu et al., 2019; Durante et al., 2019; Patterson et al., 2019), the substantia nigra (Masuda-Suzukake et al., 2013; Harms et al., 2017) and the cortex (Luk et al., 2012b). Similarly, the inoculation of purified brain extracts from PD patients or transgenic mice containing pathological α-Syn has been performed in rodents and non-human primates (Luk et al., 2012b; Recasens et al., 2014). Most of these models have succeeded in producing the accumulation and aggregation of phosphorylated α-Syn (pα-Syn), the progressive degeneration of dopaminergic neurons, a significant reduction in striatal dopamine levels, neuroinflammation, and the development of motor deficits (Recasens et al., 2014; Paumier et al., 2015; Karampetsou et al., 2017; Shimozawa et al., 2017; Patterson et al., 2019). It is well described that intracerebral inoculation of PFFs leads to the formation of pα-Syn-immunoreactive aggregates that are distributed both in the soma and neuronal processes. α-Syn inclusions exhibit different morphological features that resemble human Lewy bodies: from granules with cytoplasmic staining to compact and rounded structures with dark staining that fills entirely cells (Luk et al., 2012a; Paumier et al., 2015; Chu et al., 2019). Some studies have shown that these aggregates commonly colocalize with key markers of Lewy bodies, including ubiquitin and p62/proteasome 1 (Wakabayashi et al., 2013), and they are thioflavin S-positive and proteinase K-resistant, indicating that they share common properties with human Lewy bodies (Paumier et al., 2015; Chu et al., 2019). Our group has observed the presence of pα-Syn-immunoreactive structures with similar morphological characteristics at 12 weeks after intrastriatal inoculation of human wild-type αSyn PFFs in the SNCA-OVX transgenic mouse model (Janezic et al., 2013; **Figure 1**).

**Figure 2:**
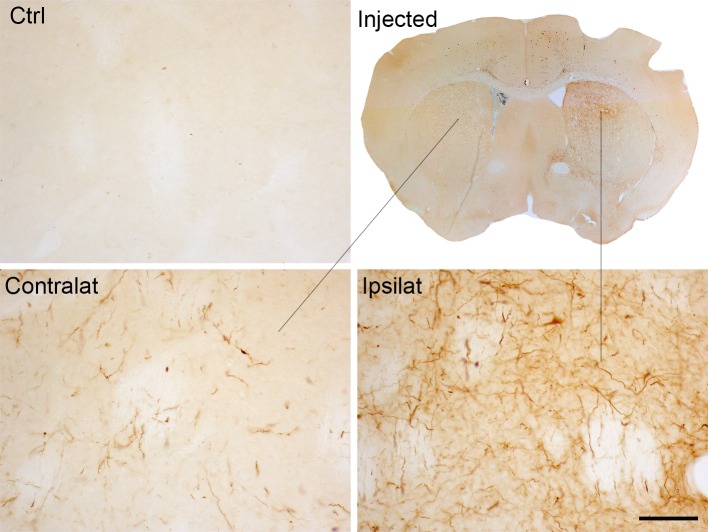
α-Syn expression in the contralateral side to the injection of α-Syn PFFs. Representative photomicrographs of pα-Syn-stained sections of the striatum of control mice or mice injected with PFFs. Scale bar: 50 µm. PFFs, pre-formed fibrils.

Also, these models have shown that pathological α-Syn can spread from the injection site to other anatomically interconnected brain regions following a fibrillary retrograde transport (**Figure 4**). The fibrils are picked up by the nerve terminals of dopaminergic neurons, travelling from them to the cell body located in the substantia nigra pars compacta. At early stages after the injection of PFFs, α-Syn inclusions are visible in regions close to the injection site, but over time the inclusions can be detected in other areas, including locus coeruleus, SNc, thalamus, hypothalamus, amygdala, and neocortex, confirming that there is a time-dependent propagation of α-Syn pathology (Luk et al., 2012a; Luk et al., 2012b; Paumier et al., 2015; Duffy et al., 2018b; Patterson et al., 2019). Some studies have reported that unilateral injection of PFFs leads to the accumulation of pα-Syn in the hemisphere contralateral to the injection (Luk et al., 2012b; Polinski et al., 2018). Indeed, we also observed small and faint pα-Syn-positive fibers into the contralateral hemisphere. These fibers are mainly found into the striatum, but also into motor cortex, amygdala and substantia nigra (**Figure 2**). The presence of α-Syn-immunoreactive inclusions in the fibers of the corpus callosum and anterior commissure, which extend bilaterally, might explain the interhemispheric transmission of α-Syn pathology.

Notably, the injection of α-Syn PFFs in transgenic null mice for murine *Snca* (i.e. α-Syn KO mice) does not lead to the formation of α-Syn inclusions or degeneration; an observation which supports that endogenous α-Syn is required for the development of the pathology (Luk et al., 2012b; Kim et al., 2019). Recasens et al. (2014) have shown that the cytoplasmic accumulation of α-Syn detected in the SNc of mice injected with Lewy body extracts at 4 months post-injection could be exclusively attributed to endogenous α-Syn, since at this time, the exogenous α-Syn could not be detected. These findings suggest that fibrils of α-Syn act as a template to convert the endogenously expressed α-Syn into pathological aggregates (Recasens et al., 2014).

Nevertheless, it is important to highlight the great variability that exists in these models because the injection site, the amount and type of PFFs injected, and the animal species/strains used might influence the development of neuropathology. Regarding the injection site, in most of the studies the α-Syn PFFs have been injected into the striatum because it is a large area with easy access. The precision required to inject PFFs is critical given that the fibrils do not spread efficiently through tissues, so adequate inoculation at the site of interest is important. Interestingly, it has been demonstrated that the injection of α-Syn PFFs into the substantia nigra of mice leads to a restriction of the pathological α-Syn expression around this brain area (Masuda-Suzukake et al., 2014) and does not induce a dopaminergic degeneration even 15 months after PFFs inoculation (Masuda-Suzukake et al., 2013). In addition, the genetic background of rodents can also influence the severity and the extent of the spreading of Lewy body-like pathology in these models. Intrastriatal injection of mouse α-Syn PFFs into different mice strains leads to differences in the severity of the α-Syn-induced pathology (Luk et al., 2012a).

Another important consideration of this model is the concentration and type of PFFs injected. Given that the *in vitro* generation of PFFs occurs under different conditions (i.e. pH, temperature, ionic strength), the sonicated α-Syn PFFs are heterogenous in nature and have different conformational features and biological effects (i.e. different aggregation pattern, propensity to propagate, seeding ability and/or toxicity). Rodents injected with different PFFs strains manifest different pathological severity and behavioral phenotypes, reflecting the great variability of the results, especially among different research studies (Peelaerts et al., 2015; Polinski et al., 2018; Chung et al., 2019). A recent study in which α-Syn PFFs have been injected into the mouse gastric wall shows that mice developed α-Syn-immunoreactive aggregates in the dorsal motor nucleus at 45 days post-inoculation, but no propagation of pα-Syn aggregates beyond of the dorsal motor nucleus of the vagus nerve was observed 12 months after inoculation (Uemura et al., 2018). These findings support that each α-Syn strain has a different potency for inducing the propagation. In addition, the animal species from which the α-Syn is derived seems to be critical. Although human and mouse α-Syn share 95% sequence identity, their ability to induce the formation of the inclusions seems to differ. Injection of mouse PFFs into the duodenal and pyloric muscularis layers of mice leads to the pathological accumulation of α-Syn in the SNc at 3 months post-injection, while the injection of human PFFs does not cause α-Syn accumulation in the SNc at the same time point (Kim et al., 2019). Moreover, previous studies have shown that human PFFs injection leads to slower propagation of α-Syn pathology compared to the injection of mouse PFFs (Rey et al., 2016). According to this finding, the formation of aggregates is more efficient if the PFFs and endogenous α-Syn are from the same species (Fares et al., 2016). Moreover, some studies have used modified forms of α-Syn PFFs, for example phosphorylated S129 fibrils or N- and C-terminally truncated forms. Mice injected with phosphorylated PFFs exhibited more α-Syn inclusions in the SNc than mice injected with wild type α-Syn PFFs (Karampetsou et al., 2017).

According to the Braak hypothesis, the PD pathology is initiated in two independent sites: the gastrointestinal tract and the olfactory bulb (Braak et al., 2003). To address the need to understand the mechanisms by which the pathologic α-Syn spread through the brain from these two starting sites, α-Syn PFFs have been administered through peripheral routes (i.e., intramuscularly and intravenously), and in the olfactory bulb. Recent studies demonstrate that the injection of α-Syn PFFs in the muscular layers of pylorus and duodenum produces α-Syn aggregation and a significant progressive degeneration of dopaminergic neurons. The accumulation of pα-Syn in the SN at 7 months post-injection coincides with a reduction of TH- and Nissl-positive cells in this region. In contrast, at 1 and 3 months after injection, there is no significant loss of TH- or Nissl-positive cells. Accompanying the loss of dopaminergic neurons, a reduction of tyrosine hydroxylase immunoreactivity in the striatum is observed at 7 months and, much more at 10 months after PFFs injection, coinciding with the appearance of pα-Syn aggregates in this area. Also, high-performance liquid chromatography analysis to measure dopamine concentration indicates that striatal dopamine levels are significantly reduced at 3 months post-injection (Kim et al., 2019). These mice develop motor, and non-motor PD symptoms, including psychiatric behavioral and olfactory dysfunction (Kim et al., 2019) and gastrointestinal dysfunction (Challis et al., 2020). In addition, these studies demonstrated that the vagus nerve and endogenous α-Syn are required for the gut-to-brain transmission of pathologic α-Syn because truncal vagotomy was performed prior to inoculation of PFFs (Uemura et al., 2018; Kim et al., 2019) and the PFFs injection in the *Snca* null mice (Kim et al., 2019) prevents the spread of pathologic α-Syn to the brain. Similarly, another recent study has shown that intrastriatal injection of α-Syn PFFs induces the Lewy body-like pathology within the enteric nervous system of wild type mice (Earls et al., 2019). Likewise, other studies have shown that nigral overexpression of rAAV-α-Syn in rats produces concomitant alterations on the enteric nervous system—accumulation of α-Syn deposits (Ulusoy et al., 2017) and a loss of enteric neurons and changes in the gut microbiome (O'Donovan et al., 2020), which reflects that α-Syn pathology in the brain may impact on the gastrointestinal system and that the gut-to-brain communication via the vagus nerve may underlie the path of pathology progression. In other studies, α-Syn PFFs have been injected into the olfactory bulb, which produces an accumulation of pα-Syn in different areas distant to the injection site, which shows that there is a spatial and multi-synaptic progression of α-Syn pathology over time. Moreover, those studies showed degeneration and olfactory dysfunction (Rey et al., 2016; Rey et al., 2018), so these models can be used to reproduce prodromal PD and test therapies designed to slow or halt the development of PD.

Given that idiopathic PD is not strictly associated with an increase in the α-Syn levels, as opposed to PD associated with duplications and triplications of the *SNCA* gene, an environment in which the levels of endogenous α-Syn are physiological would more faithfully recapitulate the non-genetic forms of PD (Duffy et al., 2018a). The α-Syn PFFs models, in contrast to those based on overexpression of α-Syn (viral vector-based and transgenic models), represent an approach in which the pathology is induced in a context of physiological levels of endogenous α-Syn (Chung et al., 2019). Thus, these models are especially useful for assessing the effect of pathological α-Syn injected exogenously on the aggregation of endogenous α-Syn, and neuronal function, and to evaluate the resultant parkinsonian phenotype. Moreover, PFFs models are considered as valuable tools to develop and test therapies based on preventing the formation of α-Syn inclusions and their spreading at the early stages of the disease. While common features are well reproducible among the different groups working on this model, a certain degree of variability still exists between the results. Establishing standard protocols for the preparation of PFFs can improve the reproducibility of the results obtained. Additional experiments would be helpful to understand the best conditions to achieve the most efficient and robust phenotype (e.g. concentrations of PFFs, site of injection, and comparison across animal species and strains). Another important consideration when using the α-Syn PFFs model is to established whether or not recombinant α-Syn PFFs are identical to the species of α-Syn present in the pathology of human PD patients (Polinski et al., 2018).

### Overexpression of α-Syn Mediated by rAAV

Another alternative to model PD is the α-Syn overexpression by recombinant adeno-associated virus vectors (rAAV). rAAV are an efficient vehicle for gene delivery in the brain area of interest and offer some characteristics that favor their use in modeling PD. rAAV efficiently transduce various cell types, confer long-lasting transgene expression, and can transduce dividing and non-dividing cells in the absence of an immune reaction (Pignataro et al., 2018). Given that neurons are post-mitotic cells, the capacity of rAAV to transduce non-dividing cells is crucial in the context of neurodegenerative disease. rAAV are smaller particles than lentivirus, which gives them the advantage of spreading efficiently within tissues, being a good choice for tissue infection. Another advantage of their small size is that many more rAAV-viral particles can be injected in the same volume compared to lentiviral particles, resulting in a much greater functional titer per injected volume. In addition, rAAV rarely integrate into the host genome, which reduces the occurrence of mutagenesis. This is crucial since random integration of the vector into the host DNA can lead to both loss- and gain-of-function mutations that might alter cell functionality and homeostasis (Albert et al., 2017).

**Figure 3:**
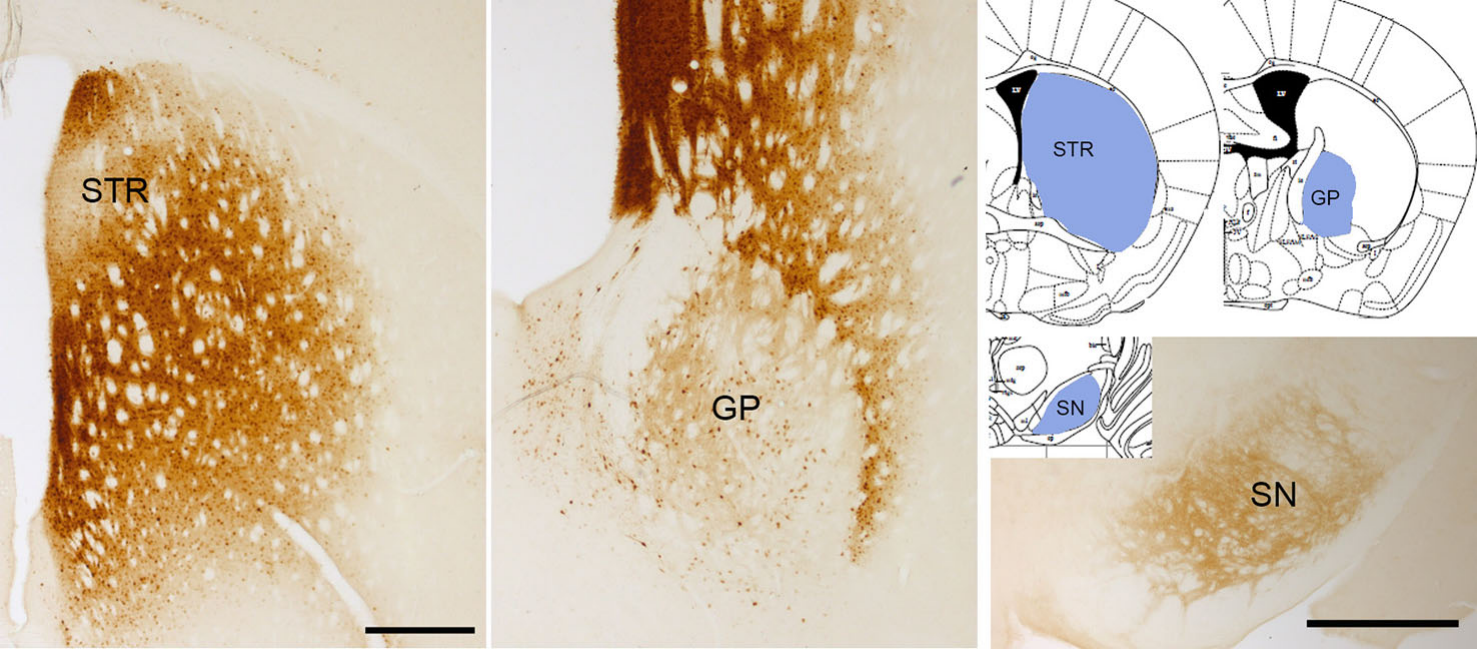
α-Syn aggregation 3 months after rAAV-E46K viral particle injection. Representative photomicrographs illustrating the expression of pα-Syn in the striatum (STR), globus pallidus (GP), and substantia nigra (SN). Scale bar: 500 µm.

**Figure 4:**
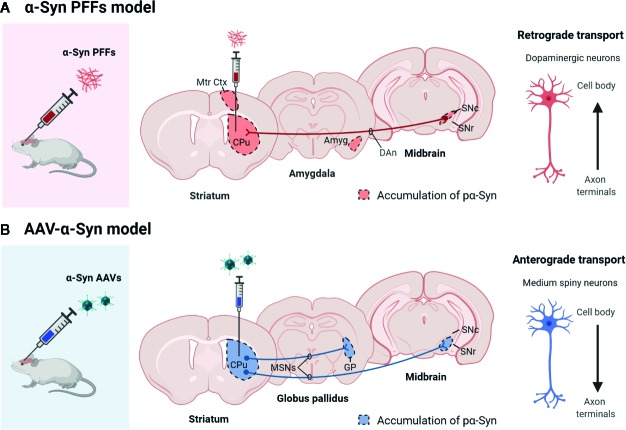
Scheme showing the propagation pattern of α-Syn in PFFs and AAV models. The colored areas represent the brain areas where we find pα-Syn expression after **(A)** α-Syn PFFs inoculation or **(B)** AAV-α-Syn administration. Created with: BioRender.com

Overexpression of wild type α-Syn or PD-associated mutants (A53T or A30P α-Syn) utilizing rAAV leads to a progressive loss of dopaminergic neurons in the SNc, a loss of dopamine terminals in the striatum (Koprich et al., 2010; Koprich et al., 2011; Oliveras-Salvá et al., 2013; Bourdenx et al., 2015; Caudal et al., 2015; Lu et al., 2015; Ip et al., 2017), and a reduction of striatal dopamine content (Koprich et al., 2011; Ip et al., 2017). However, the extent of neurodegeneration achieved with the rAAV model is variable among the different studies. Several serotypes, promoters, α-Syn species, doses, and time-course after injection have been tested, and all these factors influence the parkinsonian phenotype achieved. rAAV-α-Syn expression leads to the accumulation of pα-Syn. Unlike models based on the administration of α-Syn PFFs, in these models, the α-Syn-immunoreactive structures are commonly nuclear with a small and punctate appearance. Some studies have demonstrated that these structures are proteinase-K resistant (Koprich et al., 2010; Taschenberger et al., 2012; Lu et al., 2015; Ip et al., 2017) or urea-resistant (Oliveras-Salvá et al., 2013), but they do not reproduce the morphological features of human Lewy bodies. Our group have found that the overexpression of E46K human α-Syn mediated by rAAV2/9 in the striatum leads to the accumulation of multiple pα-Syn-immunoreactive structures in striatal cells, most likely in medium spiny projection neurons because we observe a diffuse staining of pα-Syn in the terminals in the projection fields (the globus pallidus and substantia nigra reticulata) at 12 weeks after rAAV injection (**Figure 3**). According to previous studies (Koprich et al., 2010; Ip et al., 2017), the pα-Syn-immunopositive structures are small, with a rounded appearance. Although several transgenic mice lines expressing E46K α-Syn have been generated (Emmer et al., 2011; Nuber et al., 2018), it is the first time that E46K human α-Syn form is overexpressed by viral vectors in mice. In the cell body, α-Syn aggregates are located in the nucleus while in the projection fields are in the axon terminals. We find that pα-Syn expression is maintained within the striatal medium spiny neurons, travelling from the cell body to the terminals, with an anterograde transport, but we do not observe a transsynaptic transmission between neurons (**Figure 4**). These observations suggest that rAAV-α-Syn expression does not constitute a propagation model of α-Syn as it is observed with the PFFs model, so further investigations are needed to understand the pathological mechanism of excessive amount of α-Syn coming from external source and those that are endogenously produced.

In the rAAV-α-Syn model, the presence of pα-Syn inclusions in the nigrostriatal system is concomitant with a significant loss of nigral dopaminergic neurons and the reduction in tyrosine hydroxylase immunoreactivity in the striatum. Overexpression of wild type or A53T human α-Syn induces a progressive loss of dopaminergic neurons in the SN over time (Oliveras-Salvá et al., 2013). At 5 days post-injection, no degeneration was observed. The dopaminergic cell loss in the SN increased from 57% at 4 weeks after injection to 82% at 8 weeks for wild type α-Syn; and from 51% at 4 weeks after injection to 59% at 8 weeks for A53T α-Syn. Similar to the observation for the SN, immunohistochemical staining for TH in the striatum revealed a gradual reduction of TH expression over time (Oliveras-Salvá et al., 2013). The maximum of pα-Syn-positive cells in the SN is reached at 4 weeks post-injection when neurodegeneration begins to be evident (Oliveras-Salvá et al., 2013). However, in absence of pα-Syn aggregates, i.e. when animals are injected with empty viral particles (Koprich et al., 2010; Ip et al., 2017) or in the contralateral injection side (Oliveras-Salvá et al., 2013), neurodegeneration is not observed.

Some studies show that rAAV-α-Syn expression causes the development of motor alterations, such as an increased apomorphine or amphetamine-induced rotation, defects in the stepping test or increased forepaw asymmetry in the cylinder test (Kirik et al., 2002; Decressac et al., 2011; Koprich et al., 2011; Decressac et al., 2012; Gaugler et al., 2012; Gombash et al., 2013; Oliveras-Salvá et al., 2013; Bourdenx et al., 2015; Caudal et al., 2015; Ip et al., 2017). These motor deficits appear several weeks after injection in animals with a significant loss of dopaminergic neurons.

In the rAAV-α-Syn models, the transgene expression is dependent on the serotype, the promoter, the injection site, and the titer of rAAV. The serotype rAAV2 is the most extensively used to date, probably because its production and purification methods are well-established (Van der Perren et al., 2014). However, there are some limitations associated with the rAAV2 serotype. First, rAAV2 efficiently transduces neurons but requires high doses; second, rAAV2 can induce a weak immune response in human hepatocytes (Mingozzi and High, 2013). A novel generation of viral particles has recently been produced; these particles show higher efficiency and no immunity associated with the rAAV2 capsid. Indeed, rAAV2/1, rAAV2/5, rAAV2/6, rAAV2/7, and rAAV2/8 exhibit a higher transduction efficiency than the rAAV2 in the nigrostriatal pathway (Taymans et al., 2007; McFarland et al., 2009b; Ulusoy et al., 2012; Oliveras-Salvá et al., 2013).

The efficiency of transgene expression is also determined by the design of the vector construct, and the promoter used to control the transgene expression. The most common promoters used are hybrid cytomegalovirus (CMV), chicken β-actin (CBA), phosphoglycerate kinase (PGK), and human synapsin I (Syn-1). These promoters provide high levels of transgene expression. In addition, some studies use post-transcriptional regulatory elements to improve the transgene expression, such as woodchuck hepatitis virus posttranscriptional regulatory element or polyadenylation sequence (McFarland et al., 2009a; Koprich et al., 2010; Koprich et al., 2011; Gaugler et al., 2012; Gombash et al., 2013; Oliveras-Salvá et al., 2013; Lu et al., 2015).

Determination of viral titer after its production and purification is a critical factor in defining the transduction efficiency. In contrast to α-Syn PFFs models, the levels of α-Syn achieved after transduction with rAAV-α-Syn typically exceed those found in idiopathic PD or even in PD associated with *SNCA* multiplications (Duffy et al., 2018a), despite that the phenotype severity depends on the viral dose injected (Oliveras-Salvá et al., 2013). However, although high levels of α-Syn lead to a more robust phenotype, this does not adequately reflect what happens in parkinsonian patients. Additional studies are required to determine the optimal conditions (serotype, promoter, and viral titer) to improve the ability of this model to recapitulate the human PD. However, although the modification of these factors may help to potentiate the PD phenotype, considering other factors, such as the expression of endogenous α-Syn or the nature of injected α-Syn, are necessary to reproduce the complexity of human disease in rodents.

Several parameters, including the species, the strain, and the age of animals used, influence the development of pathology. Although most studies have used rats (Kirik et al., 2002; Koprich et al., 2010; Decressac et al., 2011; Koprich et al., 2011; Gaugler et al., 2012; Gombash et al., 2013; Caudal et al., 2015; Rocha et al., 2015; Thakur et al., 2017; O'Donovan et al., 2020), the model has also been adapted to mice. This opens a wide range of possibilities due to the greater availability of knockout and transgenic mice, which could be used to assess whether certain genes protect or enhance neurodegeneration. Viral α-Syn overexpression in L444P *GBA1* mice produces a greater loss of dopaminergic neurons in *GBA1* than wild type mice, suggesting that *GBA1* mutations enhance the vulnerability of dopaminergic neurons induced by α-Syn (Migdalska-Richards et al., 2017). In *PINK1* knock out mice, overexpression of α-Syn in the substantia nigra resulted in enhanced dopaminergic degeneration as well as high levels of phosphorylated α-Syn, suggesting that the loss of *PINK1* leads to an increased sensitivity to α-Syn-induced neuropathology (Oliveras-Salvá et al., 2014). The injection of rAAV-mediating human α-Syn overexpression in knock out mice for synapsin III (Syn III) shows that silencing of Syn III could prevent the α-Syn aggregation (Faustini et al., 2018). Moreover, the genetic background of animals is important in response to α-Syn overexpression. Human α-Syn overexpression produced a larger decrease of dopaminergic neurons in C57BL/6 than in other strains. Likewise, different rat strains (Sprague-Dawley and Wistar) displayed different susceptibility to hα-Syn overexpression (Bourdenx et al., 2015). A recent study shows that deficiency of CX3CR1 receptor in mice (Cx3cr1^-/-^) exacerbates the neurodegeneration and neuroinflammation induced by overexpression of A53T human α-Syn, reflecting the importance of genetic background (Castro-Sánchez et al., 2018). The age of the animals also affects the vulnerability of dopaminergic neurons to α-Syn. Overexpression of human α-Syn into old rats produced a more robust loss of dopaminergic neurons than into young rats (Salganik et al., 2015). Also, α-Syn levels increase with age in monkeys and humans (Chu and Kordower, 2007), so the parkinsonian phenotype might be aggravated in aged mice.

In general, the rAAV-α-Syn are directly injected into the SNc to transduce dopaminergic neurons of the nigrostriatal system. Taking into consideration the special characteristics of dopaminergic neurons of the substantia nigra, that are particularly vulnerable to stress (Bolam and Pissadaki, 2012), it is important to include appropriate controls to specifically determine the neuronal damage and dysfunction caused by α-Syn overexpression. It is also crucial to analyze whether the control proteins (e.g., GFP) or empty vectors are toxic to the dopaminergic neurons (Albert et al., 2017). Although the immune response produced by rAAV in the brain is minimal, the extent of the defense response after rAAV injection must be evaluated. Some studies have demonstrated that the injection of rAAV carrying GFP results in a significant loss of dopaminergic neurons (Klein et al., 2006; Koprich et al., 2011; Landeck et al., 2017). These findings demonstrate the need to use proper controls to ensure the α-Syn specificity on the degeneration of dopaminergic neurons.

One important consideration of these models is that the α-Syn pathology is exclusively restricted to neurons transduced by rAAV to express α-Syn. There is no evidence of the transmission of the pathology to other non-transduced neurons despite the expression of endogenous α-Syn. Another significant drawback is that individual stereotaxic injection of rAAV might cause a wide variety in the expression pattern of α-Syn between animals, which makes it difficult to obtain robust and reproducible results. Morever, the injections into the SNc are technically difficult, especially in the mouse brain due to its small size, so high precision in the stereotaxic procedure is required to minimize the inter-animal variability (Volpicelli-Daley et al., 2016).

Overexpression of α-Syn mediated by rAAV represents a valuable tool to induce progressive degeneration of dopaminergic neurons, accompanied by the development of α-Syn inclusions in these neurons. Although the progression of pathological changes observed in rAAV-α-Syn models is faster than in PD patients, the time-course of these changes is sufficient to observe the different stages defined in PD patients (pre-symptomatic, early symptomatic, and advantage stage), which facilitates the identification of new therapeutic targets (Decressac et al., 2012). These models are especially useful to study how α-Syn accumulation and aggregation contributes to neuronal degeneration and its consequences, such as motor or cognitive impairment. The use of these models has also been extended to develop and evaluate potential therapies aimed at reducing the aggregation of α-Syn and prevent against neurodegeneration induced by α-Syn (Decressac et al., 2013; Xilouri et al., 2013b; Rocha et al., 2015).

## Concluding Remarks

More than two decades ago, α-Syn was identified as the main component of Lewy bodies. Since then, this protein has become established as a possible diagnostic biomarker in PD and therapeutic target. Also, numerous animal models have used this protein in attempts to reproduce PD. Each animal model offers specific aspects of the pathology of human PD, although none of them reproduce all the defining pathological and clinical features of the disease. The lack of comparable phenotypes between rodents overexpressing α-Syn or rodents injected with toxic α-Syn species reflects the difficulty of reproducing PD in animal models. Therefore, a thorough knowledge of the key features of these models is essential to choose the model that best suits the scientific questions that we want to solve. Here, we show that α-Syn PFFs models are suitable for studying the prion-like behavior of α-Syn and its propagation through the brain. While viral α-Syn overexpression models are especially useful to determine the mechanisms of α-Syn-induced toxicity but do not allow the study of their prion-like behavior. Despite these differences, both models are valuable tools for identifying novel therapeutic targets and the design and evaluation of potential therapies aimed at reducing the aggregation of α-Syn to alter disease progression.

## Ethics Statement

All experimental procedures were approved by Cajal Institute´s Bioethics Committee in accordance with the guidelines of the European Union Council Directive (DC86/609/CEE).

## Author Contributions

RM conceptualized, arranged the review, and provided funding. MG-B generated the first draft, performed the experiments, and provided the figures. NG performed the experiments and supervised and approved the figures. PG-S contributed with the experiments and provided critical feedback. AM synthetized the α-Syn PFFs and provided mice injected with PFFs, and MD provided antibodies and tools for the experiments.

## Funding

This work was supported by grants from the Spanish Ministries of Science and Innovation (SAF2016-78207-R) and of Health, Consumption and Social Welfare, Instituto de Salud Carlos III (ISCIII), Centro de Investigación Biomédica en Red sobre Enfermedades Neurodegenerativas [CIBERNED] (CB06/05/00559 and PNSD2016I033) and from Ramón Areces Foundation (172275) and from Europan Union´s Horizon 2020, research and innovation program, AND-PD project, grant agreement n° 848002 to RM and from the ERA-Net Euronanomed II JTC2015-DiaSyn project through the Spanish Ministries of Innovation, Science and Universities (PCIN-2015-98) to RM and the Walloon Region (SPW, DG06, Belgium) to AM and MD (project N° n°1510352). MG-B was supported by a fellowship JAE-Intro 2019 from Consejo Superior de Investigaciones Científicas. The authors acknowledge support of the publication fee by the CSIC Open Access Publication Support Initiative through its Unit of Information Resources for Research (URICI).

## Conflict of Interest

AM is working as UCB Biopharma employee.

The remaining authors declare that the research was conducted in the absence of any commercial or financial relationships that could be construed as a potential conflict of interest.
